# Detection of heavy metal contamination in Batlama Stream (Turkiye) and the potential toxicity profile

**DOI:** 10.1038/s41598-023-39050-4

**Published:** 2023-07-20

**Authors:** Fikriye Altunkaynak, Kültiğin Çavuşoğlu, Emine Yalçin

**Affiliations:** 1 Department of Biology, Institute of Science, Giresun University, Giresun, Turkey; 2 Department of Biology, Faculty of Science and Art, Giresun University, Giresun, Turkey

**Keywords:** Cell biology, Genetics, Plant physiology

## Abstract

In this study, heavy metal pollution in Batlama stream flowing into the Black Sea from Giresun (Turkiye) province and the toxicity induced by this pollution were investigated by *Allium* test. Heavy metal concentrations in stream water were analyzed by using ICP-MS. Germination percentage, weight gain, root length, micronucleus (MN), mitotic index (MI), chromosomal abnormalities (CAs), proline, chlorophyll, malondialdehyde (MDA), antioxidant enzyme activities were used as indicators of physiological, cytogenetic and biochemical toxicity. In addition, Comet assay was performed for detecting DNA fragmentation. Anatomical changes caused by heavy metals in the root meristem cells were observed under the microscope. *A. cepa* bulbs are divided into two groups as control and treatment. The bulbs in the control group were germinated with tap water and the bulbs in the treatment group were germinated with stream water. As a result, heavy metals such as Al, Ti and Co and radioactive heavy metals such as Rb, Sr, Sb and Ba were detected in the stream water above the acceptable parametric values. Heavy metals in the water caused a decrease in germination, root elongation, weight gain, MI and chlorophyll values, and an increase in MDA, proline, SOD, CAT, MN and CAs values. Comet assays indicated the presence of severe DNA damage. In addition, heavy metals in stream water caused different types of CAs and anatomical damage in root meristem cells. As a result, it was determined that there is intense heavy metal pollution in the stream water and this pollution promotes multi-dimensional toxicity in *A. cepa*, which is an indicator organism. For this reason, the first priority should be to prevent pollution of water resources in order to prevent heavy metal-induced toxicity in water.

## Introduction

Water and water resources are essential to provide a source of nutrients and a productive environment for all organisms. In addition, water is an essential drink for vital activities of organisms such as nutrition, respiration, circulation, excretion and reproduction. Today, the rapid increase in human populations also increases the need for fresh water rapidly. Water shortage seriously threatens biodiversity in aquatic and terrestrial ecosystems. Globally, rapid population growth, climate changes and pollution cause water stress all over the world and cause increasing pressure on water resources^1^. In recent years, excessive urbanization, industrialization and population growth and excessive use of fertilizers in agricultural activities cause water pollution in many parts of the world, leading to deterioration of water quality^2^. Therefore, water pollution is one of the most important environmental problems today. If the main causes of water pollution are listed in detail, they can be listed as plastic and polyethylene bags, pesticides, fertilizers, mining, sewage wastes, domestic wastewater, eutrophication, thermal pollution, oil spills, acid rains, radioactive wastes. Most of these factors cause heavy metal accumulation in waters and lead to heavy metal pollution^3^.

Metals with a density greater than 5 g/cm^3^ are generally referred to as heavy metals. Heavy metals are ubiquitous and they have adverse effects on the environment and organisms. Heavy metals are the most common pollutants for water resources and are used as environmental monitoring factors. Water pollution caused by heavy metals is a global environmental problem. Today, waters in many parts of the world are affected by heavy metal pollution. This pollution can cause toxic effects on aquatic flora and fauna, as well as cause various health problems in organisms by entering the food chain. Generally, there are metals such as As, Cd, Hg and Pb and radioactive elements such as U, Ba, Sb and Sr in polluted waters^4,5^.

Batlama Stream is one of the important stream originating from an altitude of approximately 1.700 m in the Eastern Black Sea Mountains (Bektaş plateau) of Giresun province (Turkiye) and pouring into the Black Sea from the west of Giresun city center. It is located in Giresun province between 40° North and 38° East. The stream, which collects the waters of an area of 161.4 km^2^, is approximately 33 km long. The average flow is 4.4 m^3^/sec and the total amount of water it carries per year is 139 hm^3^/year. It is an important drinking water source of Giresun province. Particularly, some of the drinking water of Giresun city is obtained from the deep wells of this stream. The stream is exposed to sewage and agricultural wastes at many points due to the surrounding settlements. Agricultural activities, especially hazelnut cultivation, are carried out in the stream basin. Mining, livestock, pasture, forestry and hydroelectric power plants are other activities carried out around the stream. Pesticide, hydrocarbon and heavy metal pollution originating from agricultural activities are occasionally the subject of written and visual media. The wastewater of the factories located on both sides of the stream is also drained into the stream^6,7^. Today, monitoring of water pollution and its effects on organisms has become a research focus for both governments and scientists. Therefore, there is a need for continuous assessment of the quality of water resources. In this study, heavy metal pollution in Batlama stream in Giresun (Turkiye) and multidimensional toxicity caused by this pollution in *A. cepa*, a eukaryotic indicator organism, were investigated with the help of different parameters.

## Material and methods

### Sample collection

**Figure 1:**
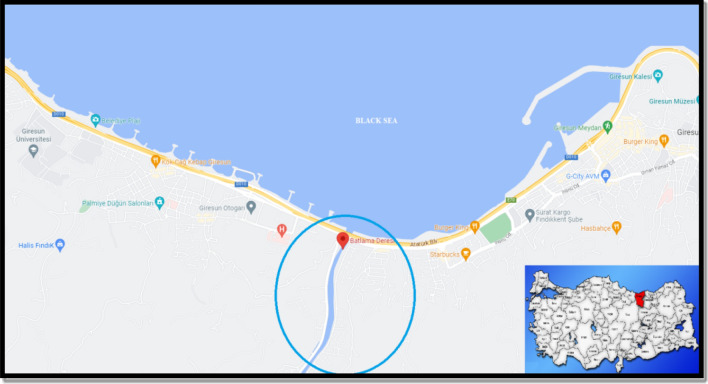
Geographical location of Batlama stream. The map showing the sampled station was created using google.com/maps-2023. The coordinates of the sampled area (GPS coordinate: 40°54′N, 38°21′E) were entered into the google map application and satellite images of the area were obtained.

This study was carried out in the Batlama stream, which flows into the Black Sea from the city center of Giresun (Turkiye). The location and coordinates of the Batlama stream, where the water samples were collected, are shown in Fig. 1. Water samples were collected on 25 September 2022. Water samples were collected from a distance of at least 100 m from the area where the stream empties into the Black Sea in order to prevent the mixing of sea water and stream water. A total of 10 water samples were taken into sterile 250 mL polyethylene bottles attached to a telescopic rod. Samples were taken at different depths in areas where there is water current and where the water stagnates.

### ICP measurements

The water samples were filtered through a 0.45 µm diameter membrane filter (Whatman Merck Millipore Corporation). 10 mL of water sample was acidified by adding 0.1 mL of concentrated HNO_3_. Quality assurance and control was carried out using triple measurement and certified reference materials (UME CRM 1201 spring water). Analytical precision was within ± 10% error. In this study, 1 ppm internal standard (Agilent 5188–6525) samples were also analyzed continuously. Heavy metal concentrations were measured using ICP-MS (Agilent Technologies 7700X Systems) in Sinop University Research Center. All measurements were made in triplicate and mean values were used.

## Experimental procedure

A. cepa (n = 16) bulbs, a eukaryotic indicator organism, were used to investigate the toxicity caused by heavy metals in water. The bulbs were divided into two groups as control and stream water treatment groups. The bulbs were placed in germination tubes after surface sterilization, and the bulbs in the control group were treated with tap water, while the bulbs were germinated with stream water in the treatment group. The solutions in which the bulbs germinated for 72 h were placed were checked every 24 h. After germination, the bulbs were used for physiological analyses and other measurements. Institutional, national and international guidelines and regulations were taken into account in the supply of plant material and the collection of water samples, and the experimental procedures carried out on the samples^8,9^.

### Physiological parameter measurements

Root elongation was determined by measuring the length of the radicle, which is the structure that forms the root in the hypocotyl and is found in the embryo of seed plants. Weight gain was determined by measuring the weights of each bulb before and after the treatment. The germination percentage was calculated using Eq. (1). 50 bulbs were used in germination percentage and weight gain test and 10 bulbs were used in root length analysis for each group^10,11^.

1 $${\text{Germination }}\left( \% \right) \, = \, \left[ {{\text{number}}\;{\text{of}}\;{\text{germinated}}\;{\text{bulbs}}} \right]/\left[ {{\text{total}}\;{\text{number}}\;{\text{of}}\;{\text{bulbs}}} \right] \, \times \, 100$$

### Chromosomal abnormality and MN detection

To determine the genotoxic effects of Batlama stream water, frequencies of CAs and MN were examined and for this purpose, 1 cm long root tips were collected from each group. Samples were fixed in Clarke's solution for 2 h and then incubated in an ethanol series. After this process, the samples hydrolyzed in 1 N HCl at 60 °C were incubated in 45% acetic acid and then stained with acetocarmine for 24 h. Samples taken between the slide and the coverslip were examined with a research microscope^12,13^. MI rates of each group were calculated with Eq. (2).

2 $${\text{MI}}\left( \% \right) = \left[ {{\text{number}}\;{\text{of}}\;{\text{cells}}\;{\text{in}}\;{\text{mitosis}}} \right]/\left[ {{\text{total}}\;{\text{number}}\;{\text{of}}\;{\text{cells}}} \right] \, \times \, 100$$

### Comet assay

DNA isolation from plant root cells was applied with the protocol suggested by Sharma et al.^14^. Comet analyses were performed according to the method suggested by Dikilitaş and Koçyiğit^15^. In summary, the slides to be used in the study were kept in ethanol for one day and made ready for use by drying in an oven. 100 μL of agarose [1% normal melting point (NMP) dissolved in distilled water at 50 °C] was placed on the slides with the help of a pipette and covered with a coverslip and layered. Slides were kept in the refrigerator for 5 min to allow the gel to freeze quickly. The second layer was formed from a mixture of 1% low melting point (LMP) agarose and freshly isolated cell suspension. The cell suspension was mixed in distilled water at 40 °C with LMP agarose at a ratio of 1/8 with a pipette on a slide, and approximately 100 µL of the mixture was spread on the first layer and immediately covered with a coverslip. The prepared preparation was left to cool for 5 min in the refrigerator and then the coverslips were removed. The preparations were immersed in the buffer solution in the electrophoresis tank and incubated for 40 min to unwind and loosen the helical DNA. Afterwards, the preparations were subjected to electrophoresis at 86 V/cm (20 V, 300 mA) for 20 min. After neutralization with tris-buffer (0.4 M Tris, pH 7.5), the slides were stained with 100 µL of ethidium bromide (0.2 mg/mL), covered with a coverslip and examined under a fluorescence microscope (Nikon Eclipse Ci-S), counted and photographed. Comet analyses were performed with the help of “TriTek 2.0.0.38 Automatic Comet Assay Software”. DNA fragments were divided into two main parts as head and tail, and the DNA amounts in these parts were expressed as %. The extent of DNA damage in the obtained Comet images was determined according to the scale proposed by Jayawardene et al.^16^. The extent of DNA damage based on numerical data was calculated according to the scale suggested by Pereira et al.^17^ considering the percentage of tail DNA.

## Biochemical parameter measurements

### MDA and proline measurements

MDA levels were measured according to the procedure recommended by Unyayar et al.^18^. 0.5 g of sample was homogenized with 5% trichloroacetic acid. Homogenate was mixed with 0.5% thiobarbituric acid and then incubated in 20% trichloroacetic acid at 96 °C. The absorbance of the supernatant obtained by centrifugation at the end of the incubation was measured at 532 nm^19^.

Proline analysis was performed according to the method suggested by Bates et al.^20^. Root tips collected from each group were homogenized with sulfosalicylic acid. A mixture of 2 mL of homogenate, glacial acetic acid and acid-ninhydrin was prepared and incubation was carried out at 100 °C for 1 h. At the end of the period, the mixture was extracted by mixing with toluene. After aspiration, the absorbance of the solution was measured at 520 nm and the proline concentration was calculated using Eq. (3).

3 $${\text{Proline }}\left( {\mu {\text{mol}}/{\text{g FW}}} \right) = \left[ {\left( {{\text{proline }}\left( {\mu {\text{g}}/{\text{mL}}} \right) \times {\text{ toluene }}\left( {{\text{mL}}} \right)} \right)/{115}.{5}} \right]/\left[ {{\text{sample }}\left( {\text{g}} \right)/{5}} \right]$$

### Leaf chlorophyll extraction and measurement

Leaf chlorophyll extraction and quantification was performed with little modification of the method proposed by Kaydan et al.^21^. 0.2 g of leaf sample was taken, cooled at 4 °C and ground by crushing with the help of a mortar. The ground samples were transferred to a tube and extracted by crushing with 5 mL 80% acetone. The mixture was transferred to a new tube, and 5 mL of 80% acetone was placed on it again and centrifuged at 3000 rpm. The filtrate was transferred to a new tube without residue and the said process was repeated once more in the presence of 80% acetone. The absorbance of the obtained green colored chlorophyll solution was determined by UV/VIS Spectrophotometer at 645 and 663 nm. Chlorophyll a and chlorophyll b concentrations were calculated using the equation of Witham et al.^22^.

### SOD and CAT measurements

Root tips of each group were collected and extracted for enzyme activities. For this purpose, 0.5 g of sample was homogenized in cold sodium phosphate buffer and the supernatant was used for biochemical analysis. SOD enzyme activities were measured according to the procedure proposed by Beauchamp and Fridovich^23^ and expressed as U/mg FW. CAT activity was measured according to the method developed by Beers and Sizer^24^ and given as OD_240nm_min/g. All analyses were performed in triplicate to determine the levels of SOD, CAT and MDA^25^.

### Observation of meristematic cell damage

Cross-sections were taken to investigate the effects of stream water exposure on root anatomy. Root samples were washed three times consecutively with distilled water to remove surface residues then cross-sections were taken from root tips collected from bulbs belonging to each group. Sections placed between the slide and the coverslip were stained with 5% methylene blue and made into a fixed preparation with Canadian balm. Anatomical structures were examined with the IM-450 TI model research microscope^26^.

### Statistical analysis of experimental data

Statistical analysis was performed in order to evaluate the significant differences between treatment group and control. Statistical analysis of the results was performed using the “IBM SPSS Statistics Version 22” program. All results are given as mean ± standard deviation (SD). Statistical significance between the values was analyzed using the “Independent Sample t-test” and a p value less than 0.05 at the 95% confidence interval was considered statistically significant. P values greater than or equal to 0.05 were considered statistically insignificant^27^.

## Results and discussion

### Heavy metal pollution

The heavy metal concentrations measured in the Batlama stream water are shown in Table 1. Among the heavy metals measured in stream water, the highest concentration was aluminum (Al). In addition, the presence of titanium (Ti), cobalt (Co) and mercury (Hg) was determined. All three heavy metals are among the heavy metals that should not be present in drinking and utility waters by the Turkish Standards Institute (TSE), the European Union (EU) and the World Health Organization (WHO). In addition, radioactive elements such as rubidium (Rb), strontium (Sr), barium (Ba), gadolinium (Gd) and uranium (U) that should not be present in drinking and utility waters were determined. Other measured heavy metal concentrations were determined to be in the maximum parametric value range for drinking and utility water determined by TSE, EU and WHO.

**Table 1:** Heavy metal and radioactive element concentrations in Batlama Stream water.

Element	Concentration (µg/L)	Maximum parametric values for drinking/potable water (µg/L)			Recovery (%)
		Turkish Standardization Institute (TSE)	European Union (EC)	World Health Organization (WHO)	
Aluminum (Al)	614 ± 49.6	200	200	200	97.81
Titanium (Ti)	5.01 ± 0.12	0	0	0	–
Chromium (Cr)	0.73 ± 0.04	50	50	50	97.05
Manganese (Mn)	7.00 ± 0.07	50	50	50	98.80
Cobalt (Co)	0.13 ± 0.01	0	0	0	99.80
Nickel (Ni)	0.30 ± 0.01	20	20	20	97.92
Copper (Cu)	1.97 ± 0.07	2000	2000	2000	99.29
Arsenic (As)	1.87 ± 0.04	10	10	10	99.32
Cadmium (Cd)	0.00 ± 0.00	5	5	3	98.31
Mercury (Hg)	0.05 ± 0.01	0	0	0	–
Lead (Pb)	0.27 ± 0.02	10	10	10	98.87
Rubidium (Rb)	1.60 ± 0.08	0	0	0	–
Strontium (Sr)	143 ± 1.14	0	0	0	99.19
Antimony (Sb)	0.29 ± 0.03	5	5	5	98.87
Barium (Ba)	37.1 ± 0.23	0	0	0	98.46
Godolinium (Gd)	0.09 ± 0.02	0	0	0	–
Uranium (U)	0.01 ± 0.01	0	0	0	–

Results are given as mean ± standard deviation. The parametric value indicates the monomer residue concentration resulting from the polymer in contact with water.

It is thought that the heavy metal (including radioactive ones) pollution measured in the water may have resulted from the household wastes located near the stream, agricultural activities carried out around the stream (especially pesticide pollution from hazelnut cultivation), mining activities and hydroelectric power plants. There are some study results in the literature that support this idea. Ghanem et al.^28^ determined heavy metal pollution such as Cd, Pb, Fe, Zn, Cr and Cu due to the use of agricultural pesticides in the groundwaters of Jenin and Tulkarem in the north of the West Bank (Palestine). Dökmeci^29^ reported the presence of heavy metal pollution, especially Pb, in the Ergene River (Turkiye) due to rapidly increasing industrialization and urbanization. Shui et al.^30^ detected the presence of heavy metal pollution in the sediments of the Gorges Reservoir (China) from agricultural chemical fertilizers, industrial wastes and ship fuel dumping. Doğan et al.^31^ observed the presence of heavy metal pollution caused by pesticides used in hazelnut farming, hydroelectric power plants and industrial establishments in Pazarsuyu stream (Turkiye).

### Physiological findings

The effects of stream water treatment on *A. cepa* physiology are shown in Table 2. 100% germination, average 7.97 cm root elongation and average 8.37 g weight gain were determined in the control group. In the group treated with stream water, 77% germination, 3.37 cm root elongation and 4.10 g weight gain were observed. Heavy metals in the stream water reduced germination by 23%, root elongation by 2.4 times and live weight by about 2 times compared to the control (p < 0.05). In the Levene test, there is a statistically significant difference between the root lengths and final weights of the control and treatment groups at the 95% confidence interval. Literature studies investigating the effects of heavy metal pollution in river waters on plants also support our findings. Çavuşoğlu et al.^32^ determined that refinery wastewaters from Kızılırmak River (Turkiye) including heavy metals caused a decrease in the growth, root elongation and weight of *Zea mays* L. (corn). In a similar study, Çavuşoğlu et al.^33^ reported that heavy metal pollution (Pb, Ni,Al, Fe, Cr, Zn, Cu, Cd) caused by the discharge of petroleum wastewater in the Melet River (Turkiye) caused a decrease in the physiological development, root elongation and weight of *Vicia faba* L. Doğan et al.^34^ observed that heavy metal pollution (Cr, Fe, V, Zn, Ni, Co, Pb, As, Cu) determined in Civil stream water (Turkiye) caused a decrease in root elongation and weight parameters of *A. cepa*.

**Table 2:** Regressions in physiological toxicity induced by Batlama stream water.

Groups and statistical analysis *Independent samples t test (univariate*)	Germination (%)	Root elongation (cm)	Weight gain (g)	Initial weight (g)	Final weight (g)
Control (*tap water*)	100	*7.97 ± 1.01	8.37	8.23 ± 0.20	*16.6 ± 0.66
Treatment (*stream water*)	77	*3.37 ± 0.67	4.10	8.20 ± 0.19	*12.3 ± 0.90
Levene's test for equality of variances (F)	–	1.911	–	0.22	1.027
Levene's test for equality of variances (Sig.)	–	0.184	–	0.882	0.324
T-test for equality of means (t)	–	11.956	–	0.393	12.357
T-test for equality of means [Sig.(2-tailed)]	–	p < 0.05	–	p > 0.05	p < 0.05
T-test for equality of means [mean difference (95% confidence interval of the difference- lower and upper)]	–	3.78 5.41	–	−0.15 0.22	3.61 5.09

All results are given as mean ± SD. Control and treatment group data were compared with each other and there was a statistically significant difference between the values indicated with * sign (p < 0.05).

It is thought that the significant decrease determined in physiological parameter values of *A. cepa* may be due to the heavy metal pollution in the stream water preventing the roots from taking up water and mineral substances, causing damage in the root cells and reducing mitotic division. Heavy metal exposure causes damage to plant root and shoot cells, as well as to vital organelles in cells such as mitochondria and chloroplasts. This prevents the uptake of water and nutrients from the roots, reduces energy production and photosynthetic rate, and affects the physiological and morphological (relatively small leaf area) structure of the plant^35^. The different types of anatomical damages observed in the root structure in our study strengthen the accuracy of this hypothesis. Singh et al.^36^ stated that the main factor of root elongation is cell division, and that exposure to heavy metals suppresses root growth by causing a decrease in the mitotic activity of root cells. In our study, a significant decrease was observed in the mitotic activity of the stem cells of the group treated with stream water.

### Cytogenetic observations

**Figure 2:**
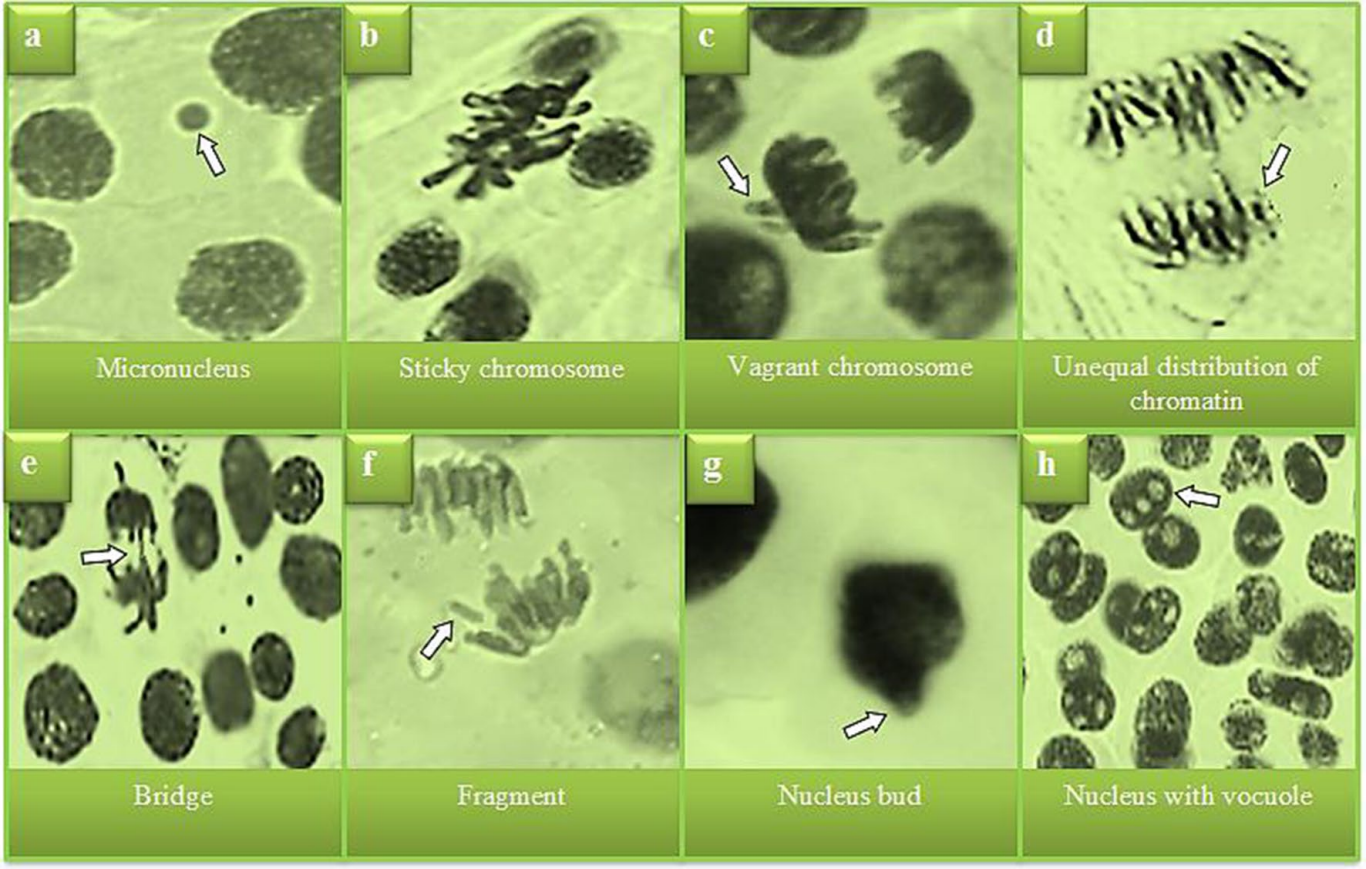
CAs induced by heavy metals from stream water. Bar: 10 µm.

The genotoxicity induced by the treatment of stream water in *A. cepa* root meristem cells is shown in Tables 3 and 4 and Fig. 2. The highest MI value of 8.61% was determined in the control group. The MI was found to be 5.40% in the group treated with stream water. Heavy metal pollution in Batlama River reduced the mitotic division by 3.21% (p < 0.05). In addition, low levels of MN and CAs were observed in the control group (p > 0.05). Stream water treatment caused an increase in MN and CAs frequencies (p < 0.05). In the Levene test, there is a statistically significant difference between the MI rates, CAs and MN frequencies of the control and treatment groups at the 95% confidence interval, but there was no statistical difference in initial weight. DNA damage caused by heavy metals was evaluated with the Comet test, which is a very sensitive and reliable analysis method. The highest head diameter, head density and head DNA were measured in the control group. Apart from slight scattering of the nucleus material, no distinctive tail was observed in the control group. While head diameter, head density and head DNA amount decreased considerably in the group exposed to stream water, tail length, tail density and tail DNA amount increased considerably. It was determined that this decrease and increase were statistically significant (p < 0.05) compared to the control group.

**Table 3:** Cytogenetic toxicity induced by Batlama stream water.

Groups and statistical analysis (*Independent samples t test*)	MI (%)	MN	SC	VC	UDC	B	FRG	NB	NV
Control (*tap water*)	*8.61	*0.30 ± 0.48	*0.40 ± 0.52	*0.30 ± 0.48	*0.10 ± 0.32	*0.00 ± 0.00	*0.00 ± 0.00	*0.00 ± 0.00	*0.00 ± 0.00
Treatment (*stream water*)	*5.40	*33.8 ± 4.10	*28.9 ± 3.60	*25.30 ± 3.80	*23.9 ± 3.90	*21.7 ± 2.71	*17.3 ± 2.21	*15.4 ± 2.12	*8.60 ± 2.12
Levene's test for equality of variances (F)	1.345	48.002	10.499	13.560	19.384	36.068	40.613	36.450	36.450
Levene's test for equality of variances (Sig.)	0.261	0.000	0.005	0.002	0.000	0.00	0.00	0.00	0.00
T-test for equality of means (t)	26.119	-25.635	-24.754	-20.627	-19.234	-25.321	-24.714	-22.985	-12.836
T-test for equality of means [Sig.(2-tailed)]	p < 0.05	p < 0.05	p < 0.05	p < 0.05	p < 0.05	p < 0.05	p < 0.05	p < 0.05	p < 0.05
T-test for equality of means [mean difference (95% confidence interval of the difference- lower and upper)]	295 346	-36.2 -30.6	-25.9 -31.1	-22.3 -27.7	-21.0 -26.6	-19.8 -23.6	-15.8 -18.9	-13.9 -16.8	-7.08 -10.1

Data are shown as mean ± SD (n = 10). 1.000 cells for MN and CAs numbers and 10.000 cells for MI were analyzed in each group. Control and treatment group data were compared with each other and there was a statistically significant difference between the values indicated with * sign (p < 0.05), there was no significant difference between the values indicated with ** sign (p > 0.05). MI: mitotic index, SC: sticky chromosome, VC: vagrant chromosome, B: bridge, NB: nucleus bud, UDC: unequal distribution of chromatin, FRG: fragment, NV: nucleus with vacuole, MN: micronucleus.

**Table 4:** DNA damage data determined by comet assay.

Parameters	Control	Stream water	Comet visuality
Head diameter (px)	26.000	10.000	
Head Intensity	91.477	17.154	
Head DNA (%)	*98.4 ± 0.47	*5.71 ± 0.35	
Tail Length (px)	0	61.000	
Tail Intensity	1.486	283.015	
Tail DNA (%)	*1.60 ± 0.47	*94.29 ± 0.35	
Tail Moment	0.070663	27.129	

Data are shown as mean ± SD (n = 10). 1000 cells for DNA damage were analyzed in each group. Head DNA and tail DNA data analyzed by Independent samples t-test.*indicates that the differences between the means are statistically significant (p < 0.05). The comet scale showing DNA damage was constructed according to the percentage of tail DNA. No or minimal damage (≤ 5%), low damage (5–20%), medium damage (20–40%), high damage (40–75%), severe damage (≥ 75%). The visual severity of DNA damage was determined by the ratio between tail and nucleus diameter. No tail (no damage), tail length equal to or shorter than the nucleus diameter (little damage), tail length less than 2 times the nucleus diameter (moderate damage), tail length equal to 2 times the nucleus diameter (major damage), tail length 2 times the nucleus diameter more than twice (maximum damage).

Our results are similar to the results of other studies investigating the genotoxic effects of heavy metal pollution in water. Barbosa et al.^35^ reported that deteriorated water quality due to heavy metal contamination in Lake Extremoz (Brazil) caused an increase in the frequency of CAs and a decrease in MI in *A. cepa*. Çavuşoğlu et al.^37^ observed that heavy metal pollution from petroleum wastewater in the Kızılırmak River (Turkiye) caused an increase in the frequency of MN in *V. faba*. Mantos et al.^38^ determined that heavy metal ions (Fe, Zn, Cr, Cu and Al) determined to be above the limit values in the Poti River (Brazil) cause a decrease in MI and an increase in CAs such as MN, nuclear bud and DNA helix breaks in *A. cepa* and *Oreochromis niloticus* L. Naf'i Al and Khalil^39^ showed that Cd, Zn, Cu and Pb exposure at 5,10, 20 and 25 ppm doses caused significant DNA damage in meristematic cells of *A. cepa* roots. Ezzat et al.^40^ observed that Cr exposure significantly increased DNA damage in *A. cepa* and *Nigella sativa* root cells. The genotoxicity (reduction in MI and increase in MN, chromosomal abnormality and DNA damage) can be associated with heavy metal ions directly or indirectly generating ROS, interacting with DNA and microtubules, and reducing mitotic activity. The information in the literature on this subject supports this idea. Jadon and Malik^41^ reported that elements such as As and Ni are messengers of mutagenic changes in the cell, and Ni causes single strand breaks in DNA and sister chromatid exchange. They also stated that transition metals cause serious damages by reacting directly with DNA and cause G → T transversions. Similarly, Wang et al.^42^ showed that Cr can directly damage DNA, cause CAs and induce mutations. Shahid et al.^43^ reported that Fe and Cu metals directly produce free radicals and as a result, they cause membrane fragmentation, lipid peroxidation, DNA damages, protein and carbohydrate abnormalities. Dutta et al.^44^ found that heavy metal-induced high-level of ROS production causes epigenetic changes, DNA damage, genetic instability and metabolic re-programming. On the other hand, heavy metals are also known to cause replication of mutated DNA by distorting the double-strand break repair process in favor of error-prone results^36^. In addition, Gzyl et al.^45^ reported that the microtubule cytoskeleton is one of the main targets of some heavy metals and that heavy metals reduce mitotic activity by disrupting microtubule arrangement, randomization and depolymerization.

### Biochemical findings

The effects of stream water treatment on the biochemistry of *A. cepa* are shown in Table 5. The lowest MDA (3.81 ratio) and proline (12.5 ratio) levels and the highest chlorophyll a (11.5 ratio) and b (4.86 ratio) levels were measured in the control group. In addition, the lowest antioxidant enzyme activities (59.0 for SOD and 0.58 for CAT) were also measured in the control group. Stream water treatment caused an increase in MDA, proline levels and antioxidant enzyme activities (p < 0.05), and a decrease in chlorophyll a and b levels (p < 0.05). Compared to the control group, stream water treatment increased MDA level approximately 2.6 times, proline level approximately 2.0 times, SOD activity approximately 1.9 times and CAT activity approximately 2.2 times. On the other hand, it decreased chlorophyll a level 1.7 times and chlorophyll b level 1.8 times. In the Levene test, there is a statistically significant difference between the biochemical parameters of the control and treatment groups (p < 0.05) at the 95% confidence interval.

**Table 5:** Biochemical alterations induced by Batlama stream water.

**Groups and** **statistical analysis** **(*Independent samples t test*)**	**MDA** **(µM/g FW)**	**Prl** **(µmol/g FW)**	**Chl a** **(mg/g FW)**	**Chl b** **(mg/g FW)**	**SOD** **(U/mg FW)**	**CAT** **(OD**_**240nm**_** min./g FW)**
Control (*tap water*)	*3.81 ± 0.36	*12.5 ± 0.96	*11.5 ± 0.83	*4.86 ± 0.62	*59.0 ± 3.36	*0.58 ± 0.05
Treatment (*stream water*)	*9.91 ± 1.09	*25.2 ± 1.76	*6.74 ± 1.08	*2.70 ± 0.36	*114 ± 5.24	*1.30 ± 0.16
Levene's test for equality of variances (F)	12.789	4.823	1.142	3.830	4.670	12.728
Levene's test for equality of variances (Sig.)	0.002	0.041	0.229	0.066	0.044	0.002
T-test for equality of means (t)	-16.799	-20.066	11.064	9.546	-27.882	-13.856
T-test for equality of means [Sig.(2-tailed)]	p < 0.05	p < 0.05	p < 0.05	p < 0.05	p < 0.05	p < 0.05
T-test for equality of means [mean difference (95% confidence interval of the difference- lower and upper)]	-5.30 -6.90	-11.4 -14.1	3.84 5.65	1.68 2.64	-50.7 -59.1	-0.60 -0.83

Data are shown as mean ± SD (n = 10). Control and treatment group data were compared with each other and there was a statistically significant difference between the values indicated with * sign (p < 0.05), there was no significant difference between the values indicated with ** sign (p > 0.05). MDA: malondialdehyde, Prl: proline, SOD: superoxide dismutase, Chl: chlorophyll, CAT: catalase.

Our results are similar to the results of other studies investigating the effects of heavy metal pollution in water on plant biochemistry. Petrovic and Krivokapic^46^ reported that heavy metal pollution (Pb, Zn, Cu, Cd) in Skada lake (Montenegro) caused proline accumulation in the roots of *Trapa natans* L. and a decrease in leaf chlorophyll levels. Nowwar et al.^47^ observed that heavy metals (Ni, Cd, Pb, Co) in un-treated wastewater samples collected from El-Rahawy (Egypt) drainage significantly increased the proline, total phenol, SOD, CAT, peroxidase, polyphenol oxidase content of *Phaseolus* plant and stimulated lipid peroxidation. Doğan et al.^31^ determined that heavy metal pollution (Fe, Ni, Mo, Ba, Sr, Be) in Pazarsuyu (Turkiye) stream caused an increase in MDA levels and SOD and CAT activities of *A. cepa* root cells.

MDA is one of the main metabolites formed as a result of oxidation of lipids in cell membranes and deterioration of their structures. It is therefore used to assess the extent of cell damage induced by different toxic agents such as heavy metals, pesticides and chemicals. MDA induces intramolecular bindings in macromolecules such as protein and lipids by affecting ion exchange from cell membranes, and also disrupts the DNA structure and suppresses genes that play a role in the defense of the plant against stress factors^10,45^. Proline is one of the 20 amino acids that are the building blocks of proteins in organisms. Proline is a non-essential amino acid. It is an essential component of many proteins. Studies have shown that the proline content in plants increases under different stress conditions. This means that the increase in proline in plants has a protective role. Proline is the first amino acid synthesized in plants under stress. It plays a role in ensuring protein integrity in plants under stress, protecting them from the effects of osmosis and turgor pressure, and activating enzymes^48,49^. Chlorophyll is the green pigment that acts as a photoreceptor in plants. There are different forms such as chlorophyll a and chlorophyll b. Its structure consists of a central Mg ion, carbon and nitrogen atoms. It is found in plants, algae and cyanobacteria and responsible for the green color of young stems and leaves in plants. Because chloroplasts have thylakoid membranes containing chlorophyll, and thus energy and glucose are produced in the presence of CO_2_, water and sunlight^50^. SOD and CAT are the most powerful enzymes involved in antioxidant defense in cells. They take an active role in the defense developed by the cell against the attacks of ROS produced. Both enzymes work in synergistic harmony with each other. SOD catalyzes the conversion of superoxide radicals to H_2_O_2_, while CAT catalyzes the breakdown of converted H_2_O_2_ to water and molecular oxygen^9,10^.

Increases in MDA and proline levels and SOD and CAT enzyme activities in *A. cepa* root can be explained by the increase in ROS production after stream water treatment. The resulting ROS can cause damage to root cells and lipid destruction in cell membranes, leading to an increase in MDA levels. Root meristem cells may also have increased the synthesis of proline, SOD and CAT to protect from the effects of ROS and initiate the ROS detoxification process. Different types of cellular damage observed in the anatomy of the root of the *A. cepa* under the microscope also support this idea. On the other hand, the results of some studies carried out on this subject in the literature also support our opinion. Georgiadou et al.^51^ reported that heavy metals such as Cu and Zn increased ROS production in plant roots, inducing cellular damage resulting in increases in lipid peroxidation and antioxidant enzyme levels. Berni et al.^52^ stated that heavy metal ions produce ROS in high concentrations such as superoxide radical, singlet oxygen and H_2_O_2_, and these ROS react with nucleic acids, lipids, pigments and proteins, causing lipid peroxidation, membrane damage and enzyme inactivation. There may be more than one reason for the decrease in leaf chlorophyll a and chlorophyll b levels due to the heavy metals in the stream water. Heavy metals can reduce the amount of chlorophyll by causing damage to leaves, which are the organs of photosynthesis, and roots that carry micro and macronutrients, by decreasing the leaf Mg concentration, by promoting the production of ROS and by inhibiting the synthesis of enzymes involved in chlorophyll synthesis. The results of some studies in the literature confirm this idea. Manios et al.^53^ attributed the decrease in chlorophyll levels in *Typha latifolia* L. (cat tail) to an increase in chlorophyll hydrolysis due to metal accumulation. Zengin and Munzuroğlu^54^ reported that Cd causes a decrease in chlorophyll level by inhibiting the activities of enzymes involved in chlorophyll synthesis. Similarly, Chandra and Kang^55^ reported that heavy metals with high redox potential inhibit various biochemical reactions and cause disruptions in the synthesis of photosynthetic pigments. Supriatno and Rahmatan^56^ concluded that the decrease in total chlorophyll level measured in *Oryza sativa* L. (rice) exposed to Pb is related to damage in root and leaf anatomical structure. Delias et al.^57^ determined that Fe increases the oxidative stress by causing H_2_O_2_ accumulation in the leaves, which decreases the Mg concentration in the leaves and decreases the activities of the enzymes involved in CO_2_ fixation.

### Anatomical observations

**Figure 3:**
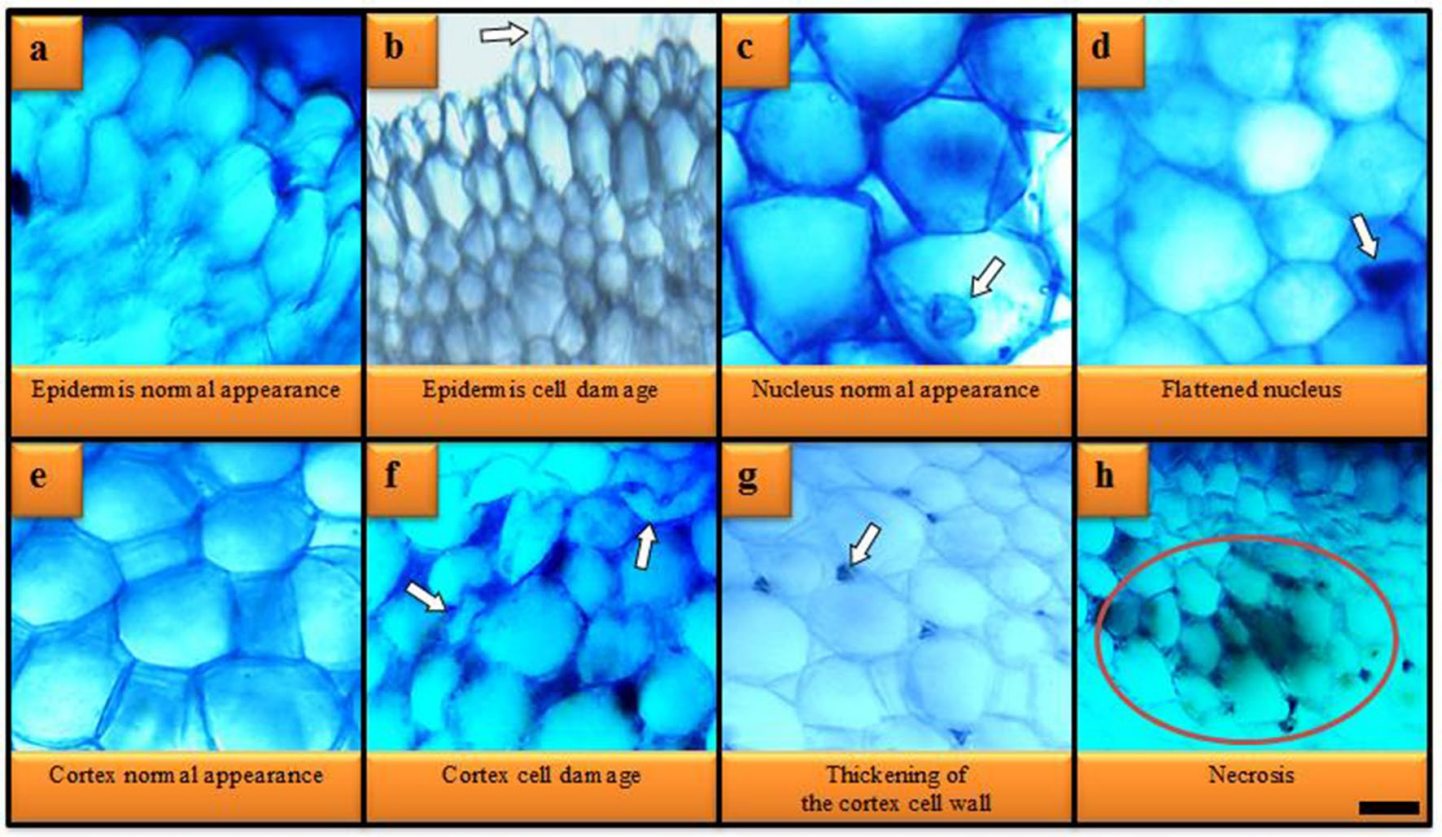
Anatomical damages induced by Batlama stream water. Bar: 10 µm.

Anatomical changes induced by stream water treatment are shown in Table 6 and Fig. 3. No damage was observed in the control group and it was observed that the root tissues were normal or healthy. In the stream water-treated group, different types of anatomical damages were observed with varying severity. Heavy metals in stream water caused meristematic cell damages shown in Fig. 3. The most severe of these damages were epidermis and flattened nucleus damages, while the lowest severity was necrosis damage.

**Table 6:** Severity of anatomical damages induced by Batlama stream water.

Groups	Epidermis cell damage	Flattaned nucleus	Cortex cell damage	Thickening of the cortex cell wall	Necrosis
Control (*tap water*)	−	−	−	−	−
Treatment (*stream water*)	+++	+++	++	++	+

(+++): severe damage, (++): moderate damage, (+): little damage, (−): no damage.

Literature studies reporting that heavy metal pollution induces anatomical damage support our findings. Türkmen et al.^58^ reported that heavy metal pollution (Pb, Al, Fe, Ni, Zn, Cu, Cd, Cr) from petroleum wastewater in the Melet River (Turkiye) caused cellular damage such as necrosis, unclear vascular tissue, flattened cell nucleus and substance accumulation in cortex cells in the root anatomy of *A. cepa*. Doğan et al.^34^ observed that heavy metal pollution (Fe, Cr, Zn, Ni, Cu, V, Co, As, Ti, Pb) in Civil (Turkiye) stream promotes damage such as necrosis, cell deformation, thickening of the cortex cell wall, unclear vascular tissue, flattened cell nucleus and substance accumulation in cortex cells in *A. cepa* root meristem cells. In a similar study, Doğan et al.^31^ determined that heavy metal pollution in Pazarsuyu stream caused anatomical damages changes such as substance accumulation in cortex cells and cell damage in *A. cepa* the root anatomy.

It is thought that the most important reason for the damages (epidermis cell damage, flattened cell nucleus and cortex cell damage) induced by stream water is the mechanical press that occurs as a result of the defense mechanism developed by the roots to prevent the entry of heavy metals into the cell. Because, under the microscope, significant increases were observed in the epidermis and cortex cell numbers of the group exposed to stream water compared to the control group. While this increase in cell numbers acts as a barrier that restricts the entry of heavy metals into the cell, it also triggers tight stacking and a mechanical pressure. This pressure can cause shape changes and deformation in the epidermis and cortex cells, as well as deformities in the nuclei of the cells. Another reason for the damage observed in root cells may be the disruption of the integrity of the cell membrane structure. Because the first affected tissue in the plant exposed to heavy metals is the root tissue. For this reason, lipid destruction can be seen in stem tissue cells under the influence of heavy metals, and deformations may occur due to disruption of membrane integrity as a result. On the other hand, the entry of heavy metals into the root cells may cause shape changes in the cell nucleus by changing the intracellular pressure, DNA volume and nuclear protein concentrations. The thickening observed in the cortex cell wall is a defense mechanism that limits the uptake of heavy metals into the cell. Excessive exposure to heavy metals causes serious damage to the plant and as a result, cell necrosis becomes inevitable. There are some study results in the literature that support our thoughts that we have stated in detail above. Emamverdian et al.^59^ reported that plants increase the synthesis of biomolecules such as proline, metallothionine and phytochelatin to protect against heavy metal toxicity. Similarly, Lapaz et al.^60^ determined that to protect from heavy metal stress of plants, they increase the synthesis of chelator compounds with protein complexes such as ferritin and nicotianamine. Yalçın and Çavuşoğlu^12^ observed that plants develop different morphological defense mechanisms, such as an increase in root cell numbers and wall thickening, to protect them from the harmful effects of heavy metals. Shahid et al.^43^ reported that induction of antioxidant enzymes, increase of phytochelatin synthesis and reduction of absorption and prevention of root immobilization are defense mechanisms developed against heavy metals.

## Conclusion

In this study, pollution by heavy metals such as Al, Ti, and Co and radioactive metals such as Rb, Sr, Sb, and Ba was detected in Batlama stream, which supplies part of the drinking and industrial water needs of Giresun province (Turkiye). Heavy metal concentrations were above the limits set by TSE, EU and WHO. This pollution is believed to be caused by industrial operations, agricultural practices and hydropower plants. The heavy metals detected in the stream water caused changes in the physiological, biochemical and anatomical structure of *A. cepa*, a eukaryotic indicator organism. Severe cytotoxic and genotoxic effects were also detected. In other words, heavy metal ions in stream water cause multi-directional toxicity. Consequently, the *Allium* test proved to be useful and reliable in determining toxicity. Considering that heavy metals in stream water reach all organisms through the food chain, it is problematic to take urgent measures. To prevent heavy metal pollution in water bodies, activities along the water sources should be controlled. For this purpose, especially settlements and industrial facilities should not be built near water resources, wastes should not be discharged into water resources and pesticides widely used in agriculture should not be mixed with water. River water should be routinely analyzed, and it should not be used for agricultural activities and drinking purposes without undergoing comprehensive treatment. High quality and clean water is the best legacy that can be left to future generations.

## Data Availability

The datasets used and/or analyzed during the current study are available from the corresponding author on reasonable request.
